# Hand grip strength and associated factors in non-institutionalised men and women 50 years and older in South Africa

**DOI:** 10.1186/1756-0500-7-8

**Published:** 2014-01-07

**Authors:** Shandir Ramlagan, Karl Peltzer, Nancy Phaswana-Mafuya

**Affiliations:** 1 HIV/STI and TB (HAST) Research Programme, Human Sciences Research Council, Pretoria, South Africa; 2 Department of Psychology, University Limpopo, Turfloop, South Africa; 3 ASEAN Institute for Health Development, Mahidol University, Bangkok, Thailand; 4 Office of the Deputy Vice Chancellor, Nelson Mandela Metropolitan University, Port Elizabeth, South Africa

**Keywords:** Hand grip strength, Social factors, Health, Behaviour, Gender, Older adults, South Africa

## Abstract

**Background:**

Little is known about the prevalence, predictors and gender differences in hand grip strength of older adults in Africa. This study aims to investigate social and health differences in hand grip strength among older adults in a national probability sample of older South Africans who participated in the Study of Global Ageing and Adults Health (SAGE wave 1) in 2008.

**Methods:**

We conducted a national population-based cross-sectional study with a sample of 3840 men and women aged 50 years or older in South Africa. The questionnaire included socio-demographic characteristics, health variables, and anthropometric measurements. Linear multivariate regression analysis was performed to assess the association of social factors, health variables and grip strength.

**Results:**

The mean overall hand grip strength was 37.9 kgs for men (mean age 61.1 years, SD = 9.1) and 31.5 kgs for women (mean age 62.0 years, SD = 9.7). In multivariate analysis among men, greater height, not being underweight and lower functional disability was associated with greater grip strength, and among women, greater height, better cognitive functioning, and lower functional disability were associated with greater grip strength.

**Conclusions:**

Greater height and lower functional disability were found for both older South African men and women to be significantly associated with grip strength.

## Background

Loss in muscle mass is a prevalent health condition associated with increasing chronological age [1,2]. Loss of muscle mass invariably results in decreased muscle strength, i.e. weakness, which is reflected in deteriorating function tests [2]. Measurement of hand grip strength can be used as indicator of muscle function. Handgrip strength (HGS) is used as a proxy measure of physical health and muscle function [3] particularly among older people. It has been used in various disciplines including in gerontological and epidemiological studies [4,5].

The determinants of HGS are reported to include age and gender in healthy people [6]. In a sample of 27,351 men and women aged 50 years and older in 11 European countries, the mean maximum HGS was 41.26 kgs for men and 24.87 kgs for women [7]. In a study of Japanese-American men aged 50–68 years old, mean maximum HGS was reported as 36.65 kgs [8]. Further, HGS has been found to be associated with functional limitations [9] and disability [1,5,7,8,10], frailty [1,8], morbidity and mortality [3,9,11,12], cognitive performance [3,9,13], inactivity, depression and self-rated health [7]. In addition, it was found that height and weight were associated with HGS [7].

Little is known about the prevalence, predictors and gender differences of HGS of older adults in Africa. This study aims to investigate social, health and gender differences in HGS among older South Africans who participated in the Study of Global Ageing and Adults Health (SAGE wave 1) in 2008.

## Methods

### Sample and procedure

We conducted a national population-based cross-sectional study with a sample of 3840 men and women aged 50 years or older in South Africa in 2008. The SAGE sample design entails a two-stage probability sample that yields national and sub-national estimates to an acceptable precision at provincial level, by locality type (urban and rural), and by population group (including African Black**, C**oloured, Indian or Asian and White). The individual response rate among those aged 50 years or older was 77%. SAGE was carried out in South Africa in partnership with the World Health Organization (WHO), the National Department of Health (NDOH), and the Human Sciences Research Council (HSRC). The study was approved by the HSRC Ethics Committee and the NDOH. Written informed consent was obtained from study participants.

### Measures

#### *Hand grip strength*

One grip test was conducted for each hand.^a^ The respondent repeated the grip exercise twice for each hand, and the better of the two measurements was recorded. The grip strength is measured in mean maximum hand grip strength (kilograms)*.* Those who had had any surgery on both their left and right arm, hand or wrist in the last 3 months or had arthritis or pain in both their left and right hand or wrist were excluded from the test. The following steps were followed to take grip strength measurements [14], p. 43: “1. Set the dynamometer to zero (0); 2. Check the fit of the dynamometer to the respondent’s hand - adjust by turning the handle to move it up or down - so that the bar should rest on the middle piece (phalanx) of the index and ring finger; 3. Ask respondent to use her/his left hand to grab the two pieces of metal, keep the upper arm close to her/his body and hold her/his forearm at right angles to the upper arm; 4. When ready, ask respondent to squeeze the dynamometer as hard as they can for a few seconds; 5. Read the dial at eye level and record strength in kilograms, rounding down to the nearest kilogram. Record ‘00’ wherever an attempt was not made; 6. Set the dynamometer to zero (0) and repeat the test with the left hand; and 7. Repeat steps 2 to 6 for the opposite hand”.

#### *Cognitive capacity*

A battery of cognitive tests was used to measure cognitive performance, in order to measure objective indicators of various aspects of cognition. The cognitive tests measured concentration, attention and immediate memory and included verbal recall, verbal fluency (a test of executive function) and digit span (forward and backward). Verbal fluency involved asking respondents to produce as many words as possible in a given category (e.g., names of animals) within a fixed time (i.e., in a one-minute time span). This test measured the ability to retrieve information from semantic memory. The variables of interest were the number of correctly named animals [15]. In terms of immediate and delayed verbal recall, the person administering the test presented 10 words verbally and repeated the words three times to saturate the learning curve. After about 10 minutes, the respondent was asked to recall as many of the 10 words as possible, to test delayed recall and recognition. Thus, verbal recall scores indicate the average number of words recalled out of the 10 words presented. This test assessed learning capacity, memory storage and memory retrieval [16]. Digit span included the forward and backward tests. For the forward test, participants read a series of digits (for example, “8, 3, 4”) and had to immediately repeat them back. If they recalled the numbers correctly, they were given a longer series of digits, until failure. In the backward test, the person was asked to repeat the numbers read to them, but in reverse order. The length of the longest list a person can remember in this fashion was that person’s digit span and was viewed as an estimate of working memory [17,18]. To test overall cognition, the following tests were selected – word list recall, verbal fluency and digit span. These accurately measure the cognitive domains most affected by impairment and the early stages of dementia. The overall cognitive score consisted of a summation of the results, and was converted to a scale of 0 to 100, where 0 represented worst cognitive functioning and 100 best cognitive functioning. The overall cognitive score was dichotomised by using the median into 48 or more and less than 48.

*Functional ability* was measured by the 12-item WHO Disability Assessment Schedule, version 2 (WHODAS-II) [19], designed to measure disability from responses to questions on physical functioning in a range of activities of daily living (ADLs) as well as instrumental activities of daily living (IADLs). Respondents were asked about difficulties in performing ADLs such as standing, taking care of household responsibilities, learning a new task as well as IADLs like getting dressed and participation in community activities in the last 30 days. Responses to these questions were scored using a five-point Likert-type response scale, ‘none’, ‘mild’, ‘moderate’, ‘severe’, and ‘extreme/cannot do’. The computed WHODAS score ranged from 0–36 and was later transformed into 0–100 with 100 being severe/extreme disability [15]. WHODAS II subscales and summary indices were coded using the International Classification of Functioning, Disability and Health (ICF) disability categories [16]; namely: No problem (0%-4%); Mild problem (5%-24%); Moderate problem (25%-49%); Severe problem (50%-95%); and Extreme problem (95%-100%), and dichotomised into 25% or above = 1 and 0- < 25 = 0.

*Subjective health status* was assessed with one question: “In general, how would you rate your health today?” Response options ranged from 1 = very good to 5 = very bad. Respondents also rated their health on nine domains: affect, mobility, sleep and energy, cognition, interpersonal activities, vision, self-care, pain, and breathing [20]. The SAGE composite health score was derived from 16 responses, two questions for each domain, using a Rasch partial credit model of Item Response Theory [21] that served to generate a composite health-state score [22,23].

*Depression*. Symptom-based depression in the past 12 months was assessed based on the World Mental Health Survey version of the Composite International Diagnostic Interview [24]. The diagnosis of depression was based on the International Classification of Diseases tenth revision (ICD-10) diagnostic criteria for research for depressive episodes [25] and was derived from an algorithm that took into account respondents reporting symptoms of depression during the past 12 months [26]. Participants endorsed at least four of ten depressive symptoms lasting 2 weeks most of the day or all of the day. According to the ICD–10–DCR criterion B, at least two of the following three symptoms needed to be present: depressed mood, loss of interest and fatigability. In addition, the ones who responded affirmatively to the question, “Have you been taking any medications or other treatment such as attending therapy or counselling sessions for depression during the last 12 months?” was added to the symptom-based depression.

*Tobacco use-* Lifetime tobacco use was assessed with the question “Have you ever smoked tobacco or used smokeless tobacco?” Lifetime tobacco users were asked “Do you currently use (smoke, sniff or chew) any tobacco products such as cigarettes, cigars, pipes, chewing tobacco or snuff?” The response options were “Yes, daily”, “Yes, but not daily” and “No, Not at all”. These questions are based on the WHO Guidelines for Controlling and Monitoring the Tobacco Epidemic [27].

*Alcohol use-* Lifetime alcohol use was assessed with the question “Have you ever consumed a drink that contains alcohol (such as beer, wine, spirits, etc.)?” Response options were “Yes” or “No, never”. Lifetime alcohol users were asked about current (past month) alcohol use, and current alcohol users were asked “During the past 7 days, how many drinks of any alcoholic beverage did you have each day?” [28] Hazardous and harmful alcohol users were defined as those who consumed 10 or more alcoholic drinks a week.

*Height and weight* were measured using a stadiometer for height and a weighing scale for weight. In terms of measuring height, an area was selected where the floor was firm, flat and close to a wall. Respondents were asked to remove their footwear and stand with their backs to the selected wall and step onto the base of the stadiometer with their feet together, knees straight and their heels, buttocks, back and head against the wall. Respondents were also asked to look straight ahead with their chin slightly tucked to their chest. The stadiometer level was then stretched to the topmost point of the head and the height was recorded to the nearest 0.1 cm. In terms of weight, respondents were asked together with their footwear, to remove all outer clothing and only wear a single layer of clothing. The weighing scale was placed on the floor that was firm, flat and close to a wall should the respondent need to lean up against the wall. The scale was checked to ensure it displayed zero and if not, it was reset to display zero. The respondent was asked to step on the scale and stand still, face forward, place their arms at their side with palms facing inwards and not to hold onto anything. The weight was recorded from the scale display in kilograms to the nearest 0.1 kg [14]. Body mass index (BMI) was used as an indicator of obesity. BMI was calculated as weight in kg divided by height in metre squared. Obesity was defined as ≥30 kg/m^2^ (BMI) and underweight <18.5. kg/m^2^ (BMI).

*Physical activity* was measured using the General Physical Activity Questionnaire (GPAQ). The instrument gathered information on physical activity in three domains (activity at work, travel to and from places, and recreational activities), as well as time spent on sitting. The questionnaire also assessed vigorous and moderate activities performed at work and recreational activities. Information on the number of days in a week spent on different activities and time spent in a typical day for each activity was also recorded [29]. For physical activity, in addition to the total minutes of activity, the activity volume was also computed by weighing each type of activity by its energy requirement in metabolic equivalents (METs). The number of days and total physical activity MET minutes per week were used to classify respondents into three categories of low, moderate, and high levels of physical activity. Physical inactivity was defined as those who had low levels of physical activity; moderate and high levels of physical activity were collapsed in further analysis [29].

#### *Chronic diseases*

Chronic diseases included arthritis, stroke, angina, diabetes, chronic lung disease, asthma, depression and hypertension. They were assessed by self-report, “Have you ever been diagnosed with e.g. diabetes (high blood sugar)?”

#### *Economic or wealth status*

To estimate economic or wealth status, a random-effects probit model was used to identify indicator-specific thresholds that represent the point on the wealth scale above which a household is more likely to own a particular asset than not. This enabled an estimation of an asset ladder. These estimates of thresholds, combined with actual assets observed to be owned for any given household, were used to produce an estimate of household-level wealth status. This was used to create wealth Quintiles [30].

### Data analysis

The data were entered using CSPro and analysed using STATA Version 10. The data were weighted using post-stratified individual probability weights based on the selection probability at each stage of selection. Individual weights were post-stratified by province, sex and age-groups according to the 2009 Medium Mid Year population estimates from Statistics South Africa [31]. Weights were not normalised. Associations between the key outcome of HGS and social and health variables were evaluated by calculating beta correlation coefficients for men and women separately. Linear multivariate regression was used for the evaluation of the impact of explanatory variables for the outcome of HGS (dependent variable). All variables statistically significant at the P < .05 levels in bivariate analyses were included in the multivariate models. In the analysis, weighted percentages were reported. Both the reported 95% confidence intervals and the P value were adjusted for the multi-stage stratified cluster sample design of the study.

## Results

### Descriptive results

The total sample included 3840 men and women of 50 years or older South Africans, 44.1% men and 55.9% women. The most prevalent population group was African Black (74%); almost half (49.9%) was between 50 to 59 years old. The educational level of most participants (71.6%) was lower than secondary school education and almost two-thirds (64.9%) lived in an urban area. Almost half (46.7%) of older adults were obese and 77.3% had hypertension, and 9.2% had diabetes. In addition, 4.0% had had a stroke, 5.2% angina, 4.9% asthma, 4% depression, 24.7% arthritis and 8.9% a nocturnal sleep problem. More than half (60.5%) engaged in low physical activity, 20.4% were daily tobacco users, and a small proportion (3.7%) was hazardous or harmful alcohol users. The mean overall HGS was 34.3 kg (mean age 61.6, SD = 6.5), 37.9 kgs for men (mean age 61.1 years, SD = 9.1) and 31.5 kgs for women (mean age 62.0 years, SD = 9.7) (see Table 1).

**Table 1:** Sample characteristics and prevalence of mean maximum grip strength among older South Africans by gender

**Variables**	**Total sample**	**Mean maximum grip strength (kg)**^**1**^	
***Sociodemographics***	N (%)	Male	Female
		M (SD)	M (SD)
*All*	3840	37.9 (19.6)	31.5 (17.8)
*Age in years (M, SD)*	61.6 (9.5)	61.1 (9.1)	62.0 (9.7)
50–59	1695 (49.9)	40.4 (19.6)	32.9 (18.3)
60–69	1233 (30.6)	35.8 (19.6)	31.8 (17.9)
70–79	661 (14.0)	35.0 (19.2)	28.7 (16.5)
80 and over	251 (5.5)	31.1 (16.8)	25.2 (14.2)
*Population group*			
African Black	2053 (74.0)	38.9 (19.6)	31.2 (16.4)
White	269 (9.3)	46.5 (22.3)	39.4 (22.2)
Coloured	655 (12.8)	34.5 (21.0)	28.8 (19.9)
Indian or Asian	307 (3.8)	33.0 (20.1)	31.2 (16.3)
*Educational level*			
No schooling	854 (25.2)	37.0 (17.4)	30.0 (17.2)
Less than primary	803 (24.0)	39.3 (23.2)	30.2 (17.6)
Primary	779 (22.4)	33.8 (17.5)	31.2 (16.9)
Secondary	923 (28.3)	41.0 (20.6)	35.5 (19.1)
*Wealth*			
Low	1482 (40.6)	37.2 (19.6)	29.7 (16.7)
Medium	731 (18.2)	39.2 (19.0)	31.4 (16.3)
High	1608 (41.2)	38.0 (19.7)	33.6 (19.7)
*Geolocality*			
Rural	1276 (35.1)	37.5 (19.5)	31.0 (16.3)
Urban	2561 (64.9)	38.1 (19.7)	31.8 (18.6)
***Health variables***			
Height (cms) [M (SD)]	158.5 (12.4)		
Underweight	184 (4.3)	28.8 (19.6)	32.6 (20.5)
Obese	1539 (46.7)	37.4 (19.8)	31.3 (17.7)
Cognitive functioning	1793 (52.0)	40.9 (20.3)	36.2 (19.0)
*Self-rated health*			
Very good/good	1460 (37.9)	40.8 (18.9)	34.8 (19.3)
Moderate	1681 (44.6)	37.1 (20.2)	30.1 (16.6)
Bad/very bad	617 (17.5)	31.9 (18.3)	28.1 (16.7)
*Functional disability*			
Low	1381 (34.5)	43.0 (20.4)	37.7 (20.9)
Medium	1142 (28.3)	37.0 (16.0)	30.8 (15.4)
High	1317 (37.2)	31.3 (19.2)	27.1 (15.4)
Chronic conditions (≥2 or more)	780 (21.5)	30.4 (18.5)	28.3 (17.7)
Depression	160 (4.0)	33.7 (21.0)	29.4 (20.0)
Daily tobacco use	810 (20.4)	39.4 (22.3)	31.4 (19.0)
Alcohol use (10 drinks or more a week)	158 (3.7)	41.6 (19.1)	23.2 (13.8)
Physical inactivity	2455 (60.5)	37.1 (20.0)	31.0 (18.3)

^1^294 (7.5%) were excluded from the sample since they reported to have had surgery on both their left and right arm, hand or wrist in the last 3 months or had arthritis or pain in both their left and right hand or wrist were excluded from the test.

Table 1 also shows that as age increased, HGS decreased for both men and women. HGS for men and women 50–59 years old were reported as 40.4 kgs and 32.9 kgs respectively and for those men and women 80 years and older it is reported as 31.1 kgs and 25.2 kgs respectively. Regarding different population groups, White men and women had the highest HGS compared to all other population groups, and Indian/Asian men and Coloured women had the lowest HGS. HGS was also seen to increase as education level increased and as wealth increased but there was really no difference in HGS among South African urban and rural dwellers. Underweight men had lower (weaker) HGS than underweight women, while obese men had higher HGS than obese women. Those with better self-rated health and lower functional disability scores had a better HGS than those with poor self-rated health and higher functional disability scores (see Table 1).

### Predictors of hand grip strength

In bivariate analysis for both men and women HGS reduced significantly with age. In multivariate analysis among men, greater height, not being underweight and lower functional disability were associated with greater HGS. In multivariate analysis among women, greater height, better cognitive functioning, and lower functional disability were associated with greater HGS (see Table 2).

**Table 2:** Parameter estimates of the association of grip strength with sociodemographic and health variables among older South Africans by gender

**Variables**	**Men**		**Women**	
***Sociodemographics***	**Beta**	**95% CI**	**Beta**	**95% CI**
*Age*				
50–59	Reference			
60–69	-4.57 (-8.20 to -0.98)*	-2.55 (-6.10 to 1.01)	-1.14 (-3.86 to 1.58)	1.37 (-1.03 to 3.78)
70–79	-5.41 (-9.91 to -0.90)*	-3.53 (-9.38 to 2.32)	-4.26 (-7.95 to -0.56)*	-1.23 (-5.24 to 2.79)
80+	-9.32 (-17.49 to -1.15)*	-2.25 (-10.22-5.72)	-7.74 (-10.97 to -4.51)***	-2.37 (-6.60 to 1.85)
*Population group*				
African Black	Reference	---		---
White	7.62 (-1.76 to 17.00)		8.21 (-1.70 to 18.12)	
Coloured	-4.39 (-14.16 to 5.37)		-2.42 (-9.23 to 4.39)	
Indian or Asian	-5.90 (-15.15 to 3.35)		0.02 (-4.05 to 4.08)	
*Educational level*				
No schooling	Reference	---		
Less than primary	2.34 (-2.07 to 6.75)		0.26 (-1.86 to 2.38)	1.17 (-1.44 to 3.78)
Primary	-3.24 (-7.06 to 0.56)		1.25 (-1.56 to 4.06)	0.30 (-2.31 to 2.92)
Secondary	3.98 (-0.21 to 8.16)		5.51 (1.63 to 9.38)**	2.07 (-1.56 to 5.70)
*Wealth*				
Low	Reference	---		---
Medium	2.08 (-3.33 to 7.49)		1.70 (-0.89 to 4.29)	
High	0.83 (-3.74 to 5.41)		3.85 (-0.11 to 7.80)	
*Geolocality*				
Rural	Reference	---		---
Urban	0.57 (-4.34 to 5.49)		0.83 (-4.02 to 5.67)	
***Health variables***				
Height (cms)	0.19 (0.08 to 0.30)***	0.15 (0.02 to 0.28)*	0.20 (0.08 to 0.33)**	0.13 (0.02 to 0.25)*
Underweight	-9.41 (-17.86 to -0.95)*	-8.41 (-14.87 to -1.94)*	1.17 (-5.30 to 7.64)	---
Obese	-0.50 (-5.53 to 4.52)	---	-0.37 (-2.72 to 1.99)	---
Cognitive functioning	7.61 (3.99 to 11.22)***	2.84 (-0.30 to 5.97)	8.96 (5.34 to 12.58)***	6.40 (3.07 to 9.74)***
*Self-rated health*				
Very good/good	Reference			
Moderate	-3.71 (-8.03 to 0.62)	1.88 (-3.31 to 7.06)	-4.67 (-7.59 to -1.75)**	0.17 (-2.70 to 3.04)
Bad/very bad	-8.86 (-14.44 to -3.29)**	0.82 (-3.94 to 5.57)	-6.75 (-11.41 to -2.08)**	1.52 (-3.06 to 6.10)
*Functional disability*				
Low	Reference			
Medium	-5.95 (-9.04 to -2.85)***	-5.95 (-8.54 to -3.36)***	-6.93 (-10.62 to -3.24)***	-6.33 (-10.03 to -2.63)***
High	-11.69 (-15.58 to -7.80)***	-8.92 (-14.7 to -3.22)**	-10.64 (-14.90 to -6.39)***	-8.41 (-12.92 to -3.90)***
Chronic conditions (≥2 or more)	-8.87 (-14.14 to -3.60)***	-5.03 (-11.1 to 1.06)	-4.21 (-6.87 to -1.56)**	-2.91 (-6.23 to 0.39)
Depression	-4.32 (-13.36 to 4.71)	---	-2.21 (-9.31 to 4.90)	---
Daily tobacco use	2.04 (-1.34 to 5.42)	---	-0.22 (-3.69 to 3.26)	---
Alcohol use (10 drinks or more a week)	3.95 (-2.44 to 10.34)	---	-8.48 (-16.24 to -0.72)*	-7.99 (-17.77 to 1.78)
Physical inactivity	-1.65 (-5.27 to 1.98)	---	-1.53 (-5.50 to 2.44)	---

***P < .001; **P < .01; *P < .05.

## Discussion

This aim of this study is to investigate social and health differences in HGS among older South Africans. We found in agreement with other studies [1,2,4] that older men had a higher HGS than older women, and in bivariate analysis that when age increases HGS decreases.

Comparing different population groups in South Africa, white men and women had higher HGS than all other population groups (African/Black, Indian/Asian and Coloured). South African men aged 50 years and older had a lower HGS than men in 11 European countries [7] yet South African women had a higher HGS than European women. When comparing South African men’s HGS to that of Japanese-American men [8], we see that South African men have a slightly stronger HGS of just 1.25 kgs more.

HGS tends to increase as education level as well as wealth increases in both men and women [7], yet this was not found in this study. But in terms of cognitive performance, we find that in women, better cognitive functioning was significantly associated with greater HGS. In another study [13], we see that higher cognitive functioning was positively associated with HGS and subsequently better memory, attention, and processing speed.

Height was significantly associated with HGS, similar to other studies [7], meaning that taller individuals had better HGS. Being underweight was only significantly associated with lower HGS in this study in for men, yet functional disability was, for both men and women, highly associated with HGS.

### Limitations of the study

This study had several limitations. Firstly, the self-report of health variables such as depression symptoms, tobacco or alcohol use should be interpreted with caution; it is possible that measurement errors occurred. Secondly, the WHO SAGE wave 1 study utilised the non-standard approach to HGS. However, a standardised approach to HGS was published in 2011 [32], long after the study had been conducted, and should be used in future studies. Thirdly, this study was based on data collected in a cross-sectional survey. We cannot, therefore, ascribe causality to any of the associated factors in the study. However, follow up studies are planned. Finally, data were collected from older adults who were available in the household on the day of the survey. Respondents who were institutionalized (prison, hospital, care home) and not returning to the household within seven days and those who had moved more than 50 kilometers away from the study household were not included.

## Conclusion

We conclude that greater height and lower functional disability were found for both men and women to be significantly associated with grip strength. Gender differences were that among men being underweight was associated with lower HGS and among women better cognitive functions was associated with higher HGS.

### Endnote

^a^Smedley’s Hand Dynamometer, Scandidact, Oldenvej 45, and 3490 Kvistgard, Denmark.

## Competing interests

The authors declare that they have no competing interests.

## Authors’ contributions

KP was the main contributor to the conceptualization of the study. SR and NPM contributed significantly to the first draft of the paper and all authors contributed to the subsequent drafts and finalization. All authors read and approved the final manuscript.

## References

[B1] Syddall H Cooper C Martin F Briggs R Sayer AA Is grip strength a useful single marker of frailty? Age Ageing 2003 32 6 650 656 10.1093/ageing/afg111 14600007

[B2] Kallman DA Plato CC Tobin JD The role of muscle loss in the age related decline of grip strength: cross-sectional and longitudinal perspective J Geronotology 1990 45 3 M82 M88 10.1093/geronj/45.3.M82 2335723

[B3] Gallup AC White DD Gallup GG Jr Handgrip strength predicts sexual behavior, body morphology, and aggression in male college students Evol & Human Behav 2007 28 6 423 429 10.1016/j.evolhumbehav.2007.07.001

[B4] Kerr A Syddall HE Cooper C Turner GF Briggs RS Sayer AA Does admission grip strength predict length of stay in hospitalized older patients? Age Ageing 2006 35 1 82 84 10.1093/ageing/afj010 16364940

[B5] Rantanen T Guralnik JM Foley D Masaki K Leveille S Curb JD White L Midlife hand grip strength as a predictor of old age disability JAMA 1999 281 6 558 560 10.1001/jama.281.6.558 10022113

[B6] Budziareck MB Pureza Duarte RR Barbosa-Silva MC Reference values and determinants for handgrip strength in healthy subjects Clin Nutr 2008 27 357 362 10.1016/j.clnu.2008.03.008 18455840

[B7] Hairi FM Mackenbach JP Andersen-Ranberg K Avendano M Does socio-economic status predict grip strength in older Europeans? Results from the SHARE study in non-institutionalised men and women aged 50+ J Epidemiol Comm Health 2010 64 829 837 10.1136/jech.2009.088476 19884112

[B8] Rantanen T Masaki K Foley D Izmirlian G White L Guralnik JM Grip strength changes over 27 yr in Japanese-American men J Appl Physiol 1998 85 2047 2053 9843525 10.1152/jappl.1998.85.6.2047

[B9] Norman K Stobaus N Smoliner C Zocher D Scheufele R Valentini L Lochs H Pirlich M Determinants of hand grip strength, knee extension strength and functional status in cancer patients Clin Nutr 2010 29 5 586 591 10.1016/j.clnu.2010.02.007 20299136

[B10] Giampaoli S Ferrucci L Cecchi F Lo Noce C Poce A Dima F Santaquilani A Vescio MF Menotti A Hand-grip strength predicts incident disability in non-disabled older men Age Ageing 1999 28 3 283 288 10.1093/ageing/28.3.283 10475865

[B11] Griffith CD Whyman M Bassey EJ Hopkinson BR Makin GS Delayed recovery of hand grip strength predicts postoperative morbidity following major vascular surgery Br J Surg 1989 76 7 704 705 10.1002/bjs.1800760717 2765805

[B12] Rantanen T Harris T Leveille SG Visser M Foley D Masaki K Guralnik JM Muscle strength and body mass index as long-term predictors of mortality in initially healthy men J Gerontol A Biol Sci Med Sci 2000 55 3 M168 M173 10.1093/gerona/55.3.M168 10795731

[B13] Taekema DG Ling CH Kurrle SE Cameron ID Meskers CG Blauw GJ Westendorp RG de Craen AJ Maier AB Temporal relationship between handgrip strength and cognitive performance in oldest old people Age Ageing 2012 41 4 506 512 10.1093/ageing/afs013 22374646

[B14] World Health Organization (WHO) Study of Global Ageing and Adult Health (SAGE) Survey manual 2006 Geneva, Switzerland: WHO

[B15] Jastak S Wilkinson G The Wide Range Achievement Test – Revised 1984 Jastak Associates, Inc: Wilmington

[B16] Welsh KA Butters N Mohs RC Beekly D Edland S Fillenbaum G Heyman A The Consortium to Establish a Registry for Alzheimer’s Disease (CERAD). part V. A normative study of the neuropsychological battery Neurology 1994 44 4 609 614 10.1212/WNL.44.4.609 8164812

[B17] Wechsler D Manual for the Wechsler Memory Scale – Revised 1987 San Antonia: The Psychological Corporation

[B18] Wechsler D Manual for the Wechsler Adult Intelligence Scale – Revised 1981 New York: The Psychological Corporation

[B19] Ustün T Kostanjsek N Chatterji S Rehm J Measuring health and disability: manual for WHO Disability Assessment Schedule (WHODAS 2.0) 2010 Geneva, Switzerland: World Health Organization

[B20] Salomon JA Tandon A Murray CJ World Health Survey Pilot Study Collaborating Groups Murray CJ, Evans DB Unpacking health perceptions using anchoring vignettes Health Systems Performance Assessment: Debates, Methods and Empiricism 2003 Geneva: World Health Organization 401 407

[B21] Wilson M Allen DD Li JC Improving measurement in health education and health behavior research using item response modelling: introducing item response modelling Health Education Res 2006 21 1 i4 i18 10.1093/her/cyl108 17018765

[B22] Andrich D Controversy and the Rasch model: a characteristic of incompatible paradigms? Med Care 2004 42 1 1 16 10.1097/01.mlr.0000108832.39437.9f 14707751

[B23] Wilson IA Gallagher M Eichenbaum H Tanila H Neurocognitive aging: prior memories hinder new Hippocampal encoding Trends Neurosci 2006 29 12 662 670 10.1016/j.tins.2006.10.002 17046075 PMC2614702

[B24] Kessler RC Ustun TB The World Mental Health (WMH) Survey Initiative Version of the World Health Organization (WHO) Composite International Diagnostic Interview (CIDI) Intern J Meth Psychiatric Res 2004 13 93 121 10.1002/mpr.168 PMC6878592 15297906

[B25] World Health Organization (WHO) The ICD-10 classification of mental and behavioural disorders: diagnostic criteria for research (DCR-10) 1993 Geneva, Switzerland: WHO

[B26] Ayuso-Mateos JL Nuevo R Verdes E Naidoo N Chatterji S From depressive symptoms to depressive disorders: the relevance of thresholds Brit J Psychiatry 2010 196 5 365 371 10.1192/bjp.bp.109.071191 20435961

[B27] World Health Organization (WHO) Guidelines for Controlling and Monitoring the Tobacco Epidemic 1998 Geneva: WHO

[B28] World Health Organisation (WHO) Study on global AGEing and adult health (SAGE) Core SAGE data and questionnaires Retrieved at http://www.who.int/healthinfo/systems/sage/en/index2.html, accessed 10 November 2011 10.1186/1753-6561-7-S4-S1 PMC3892776 24764490

[B29] World Health Organisation (WHO) Global Physical Activity Surveillance 2009 Geneva: WHO

[B30] Chatterji S Kowal P Mathers C Naidoo N Verdes E Smith JP Suzman R The health of aging populations in China and India Health Aff (Millwood) 2008 27 4 1052 1063 10.1377/hlthaff.27.4.1052 18607041 PMC3645349

[B31] Statistics South Africa Mid-Year Population Estimates 2009 [http://www.statssa.gov.za/publications/P0302/P03022009.pdf]

[B32] Roberts HC Denison HJ Martin HJ Patel HP Syddall H Cooper C Sayer AA A review of the measurement of grip strength in clinical and epidemiological studies: towards a standardised approach Age Ageing 2011 40 4 423 429 10.1093/ageing/afr051 21624928

